# Thiourea-Grafted Graphite Felts as Positive Electrode for Vanadium Redox Flow Battery

**DOI:** 10.3389/fchem.2020.626490

**Published:** 2021-01-14

**Authors:** Shangzhuo Wu, Xin Lv, Zhijun Ge, Ling Wang, Lei Dai, Zhangxing He

**Affiliations:** School of Chemical Engineering, North China University of Science and Technology, Tangshan, China

**Keywords:** vanadium redox flow battery, graphite felts, thiourea, grafted, energy storage

## Abstract

In this paper, thiourea was successfully grafted onto the surface of acid preprocessed graphite felts [sulfuric acid-treated graphite felt (SA-GFs)] by thiol-carboxylic acid esterification. The thiourea-grafted graphite felts (TG-GFs) were investigated as the positive electrode for vanadium redox flow battery (VRFB). X-ray photoelectron spectroscopy results suggested that thiourea was grafted into the surface of graphite felts. The cyclic voltammetry showed that the peak potential separation decreased by 0.2 V, and peak currents were greatly enhanced on TG-GF electrode compared with SA-GF electrode, implying improved electro-catalytic activity and reversibility of TG-GF electrode toward VO^2+^/${\text{VO}}_{2}^{+}$ redox reaction. The initial capacity of TG-GF-based cell reached 55.6 mA h at 100 mA cm^−2^, 22.6 mA h larger than that of SA-GF-based cell. The voltage and energy efficiency for TG-GF-based cell increased by 4.9% and 4.4% compared with those of SA-GF-based cell at 100 mA cm^−2^, respectively.

## Introduction

Vanadium redox flow battery (VRFB) as energy storage system has caused more and more attention because VRFB displays some advanced characteristics, such as long cycle life, high energy efficiency (EE), and excellent electrochemical reversibility (Bhushan et al., 2019; Li et al., 2019; Xiang and Daoud, 2019; He et al., 2020; Lv et al., 2020). The electrodes play a central role where redox reactions occur (Ding et al., 2018; Ye et al., 2018). Although the commercial graphite felts can be used as electrode materials for VRFB, the electrochemical activity is not enough for practical application (He et al., 2015).

The introduction of functional groups is one of the effective surface functional treatments to improve the electrochemical properties of the graphite felts. Among the functional groups, oxygen-containing groups, such as -COOH, -OH, and C=O, have been widely studied by various methods including heat treatment (Zhang et al., 2020), acid treatment (Sun and Skyllas-Kazacos, 2010), electrochemical oxidation (Xiao-Gang et al., 2007), and microwave treatment (Wu et al., 2014). In addition, the nitrogen-containing groups also have been reported to be active toward vanadium redox reactions. Tao et al. (2012) reported a hydrothermal ammoniated treatment for graphite felt used as the positive electrode for VRFB. The introduction of the polar nitrogenous groups can facilitate the charge transfer rate between electrode and vanadium ions. He et al. (2013) added two organic additives in positive electrolyte, which provided -NH_2_ group on the surface of the graphite felt and could be employed as active sites for vanadium ion reactions. Recently, Lee et al. (2015) reported that the supercapacitor performance based on thiourea (NH_2_CSNH_2_)-grafted graphene could be greatly improved due to introducing the amine and sulfur functional groups into graphene. In addition, the electrocatalytic properties of multi-walled carbon nanotubes toward the VO^2+^/${\text{VO}}_{2}^{+}$ redox couple were also improved by surface functional treatments using thiourea as nitrogen and sulfur sources (Li et al., 2017).

In this work, we report a novel, simple, and mild method for *in situ* functionalizing graphite felt electrode by grafting thiourea for VRFB. The -NH_2_ and C-S functional groups were successfully introduced onto the surface of graphite felts. The VRFB using the thiourea-grafted graphite felt as positive electrode showed larger discharge capacity (DC) and EE.

## Experiment

### Preparation of the Electrode

Polyacrylonitrile (PAN)-based graphite felts (GFs) (thickness: 6 mm; Beijing Jinglong Carbon Technology Co., Ltd.) were pretreated with 98% sulfuric acid at room temperature for 24 h. In order to obtain thiourea-grafted graphite felts, the sulfuric acid-treated graphite felts (SA-GFs) were placed in a beaker containing 30 ml of 150 mg ml^−1^ thiourea solution, and then the beaker was kept in a water bath at 80°C for 10 h (Lee et al., 2015).

### Characterization

Morphology of samples was characterized by scanning electron microscopy (SEM, S-4800, Hitachi, Japan). X-ray photoelectron spectroscopy (XPS) was carried out (K-Alpha 1063, Thermo Fisher Scientific, UK) for characterization of the surface chemistry of samples. Raman spectra were recorded on a laser Raman spectrometer (Thermo Electron DXR, USA).

### Electrochemical Measurements

The electrochemical measurements [cyclic voltammogram (CV) and electrochemical impedance spectroscopy (EIS)] of the prepared electrode (area: 1 cm^−2^) were carried out on IM6e Zennium electrochemical workstation (Zahner Scientific Instruments, Germany) using Pt electrode and saturated calomel electrode (SCE) as the counter and reference electrodes, respectively. The electrolyte consisted of 0.1 M VOSO_4_ and 3 M H_2_SO_4_. The scan rate of CV test was 1 mV s^−1^. The frequency range of EIS was 10^−2^-10^6^ Hz.

The charge–discharge performance for TG-GF electrode was assessed in a static cell using CT2001A (LAND, Wuhan) battery test system. The cells were assembled using TG-GF and SA-GF (3 × 3 cm^2^) as positive electrode, SA-GF as corresponding negative electrode, and perfluorinated ion-exchange membrane as separator in 1.2 M V(III)/V(IV) + 3 M H_2_SO_4_ electrolyte.

## Results and Discussion

**Figure 1:**
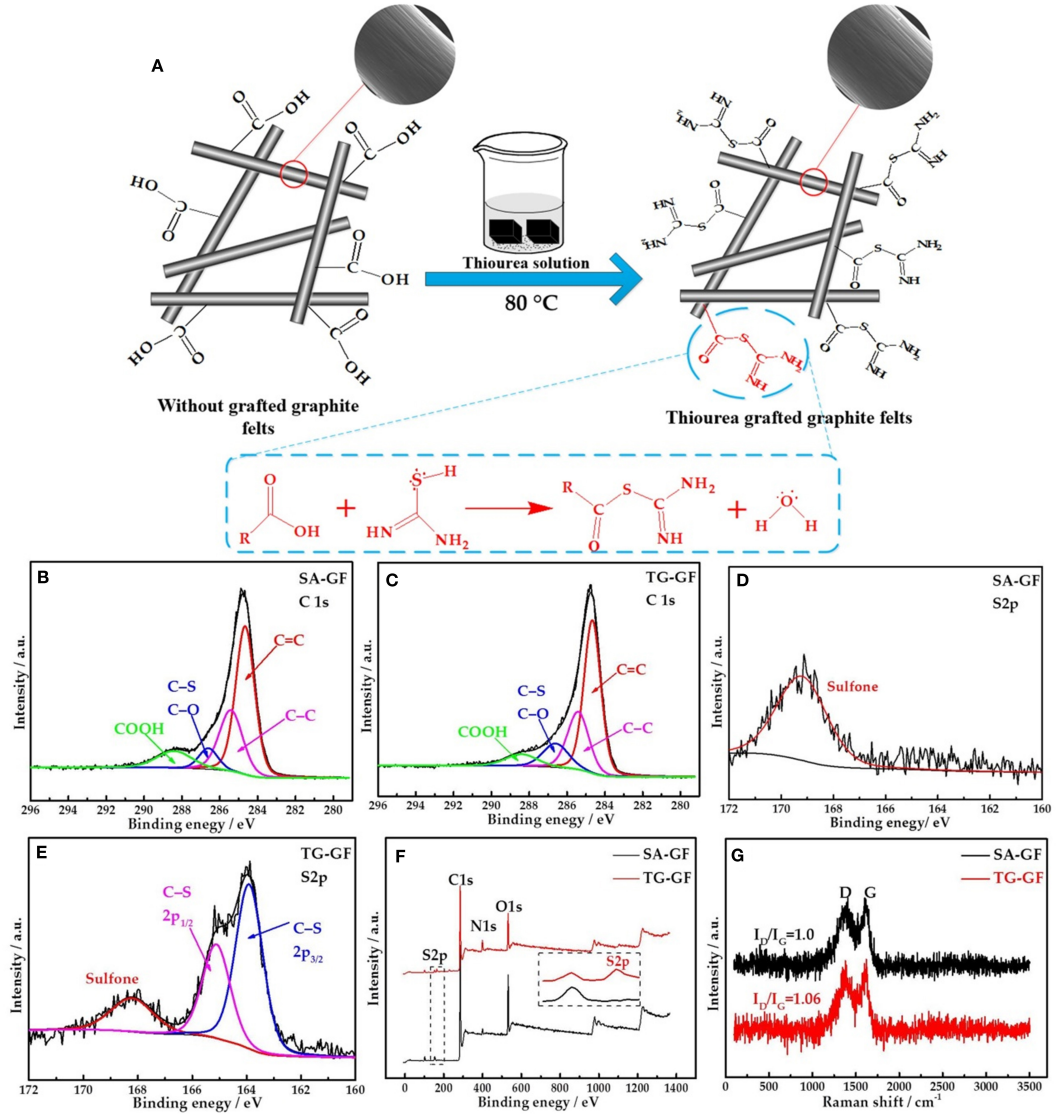
Mechanism of thiourea-grafted graphite felt (TG-GF) (including SEM photos) **(A)**. X-ray photoelectron spectroscopy (XPS) C1s spectra of sulfuric acid-treated graphite felt (SA-GF) **(B)** and TG-GF **(C)**. S2p spectra of graphite felt (GF) **(D)** and TG-GF **(E)**. Survey spectrum of SA-GF and TG-GF **(F)**. Raman spectra of SA-GF and TG-GF **(G)**.

As shown in Figure 1A, thione (C=S) in thiourea exists a resonance structure thiol (C-S-H). The amine groups connected with the C-S-H can react with carboxylic acid (COOH) on the graphite felts *via* thiol-carboxylic acid esterification (Lee et al., 2015). SEM images for SA-GF and TG-GF (Figure 1A) show no obvious change of morphology. Figures 1B,C show the C1s high-resolution XPS spectra of SA-GF and TG-GF. Two samples contain C=C (284.4 eV), C-C (285.4 eV), C-S/C-O (286.5 eV), and COOH (288.6 eV) functional groups (Liu et al., 2015; Kabtamu et al., 2017). The peak at 286.5 eV represents carbon in SA-GF bound to one oxygen or sulfur (e.g., C-O, C-S) (Gattrell et al., 2006). XPS spectra for S2p shown in Figures 1D,E indicate that TG-GF sample exhibits S2p_3/2_ and S2p_1/2_ signals at 163.9 and 165.1 eV, respectively, as well as a trace peak at 168.2 eV (Baker et al., 2004; Huang et al., 2014). As shown in Table 1, compared with SA-GF, TG-GF has more C-S groups but lower COOH functional groups, which is attributed to the reaction between the carboxyl groups on SA-GF surface and the grafting of the amine and sulfur functional groups on thiourea, accompanied by the introduction of C-S and -NH_2_ groups on the surface of SA-GF. The O atomic percentage of TG-GF decreases to 12.8% from 24.1% after grafting thiourea group onto SA-GF. Meanwhile, S atomic percentage of TG-GF increases to 1.8% from 1.1%, and N atomic percentage increases to 8.1% from 5.9%. The trace peak at 168.2 eV is ascribed to sulfone species (Huang et al., 2014; Lee et al., 2015). XPS full spectra of SA-GF and TG-GF (Figure 1F) show a very clear S2p signal appearance for TG-GF, while for SA-GF, without S2p signal. Figure 1G shows Raman spectra of both samples. SA-GF and TG-GF samples give similar Raman scattering patterns with peaks at 1,380 (D band) and 1,600 cm^−1^ (G band). The intensity ratio of D to G band (*I*_*D*_/*I*_*G*_) that represents the extent of defects in carbon materials is different. The increase of *I*_*D*_/*I*_*G*_ value from 1.00 for SA-GF to 1.06 for TG-GF means the decrease in the ordered graphite crystal structure after SA-GF grafting thiourea (Lee et al., 2015).

**Table 1:** Elemental composition and chemical composition of functional groups based on C1s and S2p XPS spectra.

**Samples**	**Elemental composition (%)**				**C1s peak deconvolution (%)**				**S2p peak deconvolution (%)**	
	**C**	**N**	**O**	**S**	**C=C**	**C-C**	**C-O/C-S**	**COOH**	**S2p_3/2_**	**S2p_1/2_**
SA-GF	68.92	5.89	24.11	1.08	55.55	23.49	11.52	9.43	–	–
TG-GF	77.22	8.1	12.83	1.85	54.33	23.56	15.75	6.35	52.68	30.59

*SA-GF, sulfuric acid-treated graphite felt; TG-GF, thiourea-grafted graphite felt; XPS, X-ray photoelectron spectroscopy*.

**Figure 2:**
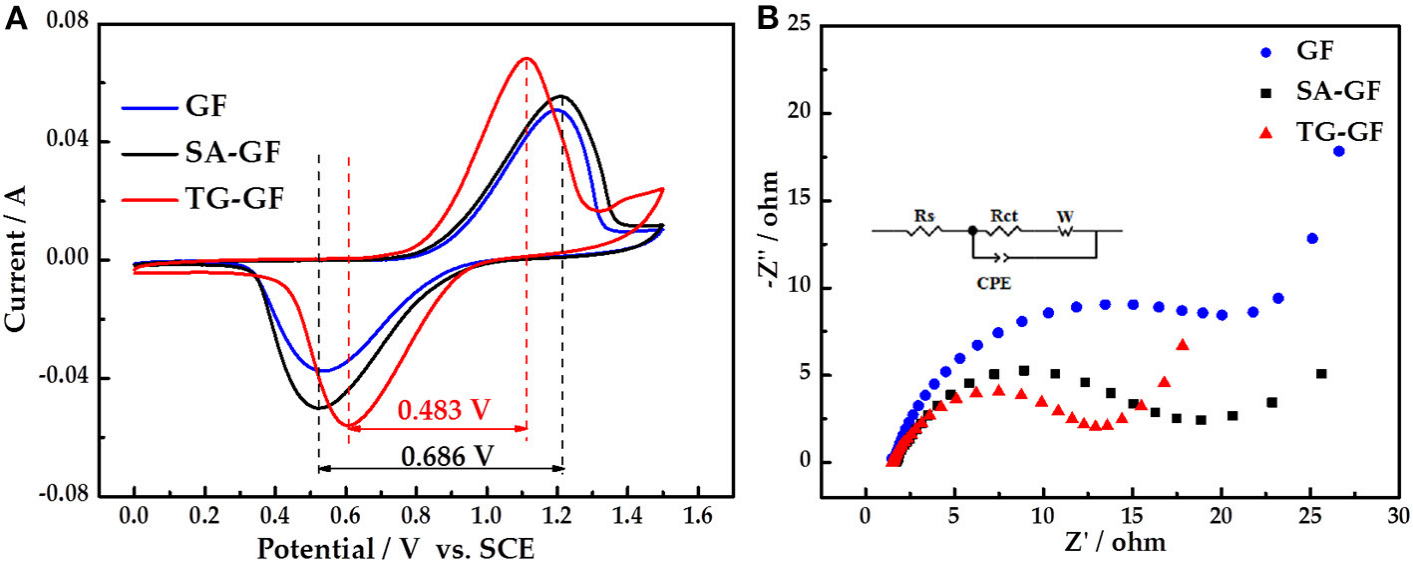
Cyclic voltammogram (CV) **(A)** and Nyquist plot **(B)** curves of graphite felt (GF), sulfuric acid-treated graphite felt (SA-GF), and thiourea-grafted graphite felt (TG-GF) in 0.1 M VOSO_4_ + 3 M H_2_SO_4_ electrolyte.

CV curves of all electrodes (Figure 2A) appear two peaks, which correspond to oxidation and reduction reactions of VO^2+^/${\text{VO}}_{2}^{+}$ couple. Compared with SA-GF, the redox peak potential separation of TG-GF dramatically decreases from 0.686 to 0.483 V. The peak currents are in the order of TG-GF > SA-GF > GF, showing that sulfuric acid pretreatment can slightly improve the performance of GF. However, thiourea grafting can greatly enhance the electrochemical activity and reversibility toward the VO^2+^/${\text{VO}}_{2}^{+}$ redox reaction. The little peak appearing at 1.3–1.5 V for TG-GF is ascribed to the slight oxygen evolution reaction.

Figure 2B shows Nyquist plots of three electrodes. A semicircle and a straight line are observed at high and low frequencies, respectively. *R*_s_ is attributed to the resistance of electrolyte and electrode. *R*_*ct*_ represents Faradaic interfacial charge-transfer resistance. The constant-phase element (*CPE*) is attributed to the double-layer capacitance, and *W* is Warburg impedance (Li et al., 2017). According to fitting results, the *Rs* values for GF, SA-GF, and TG-GF were almost equivalent. *R*_*ct*_ of GF (25.8 Ω) is higher than that of other electrodes, suggesting poorer electrochemical activity of GF. The decrease of *R*_*ct*_ value from 15.50 Ω for SA-GF to 10.25 Ω for TG-GF indicates that grafting thiourea onto SA-GF can reduce the electrochemical polarization.

**Figure 3:**
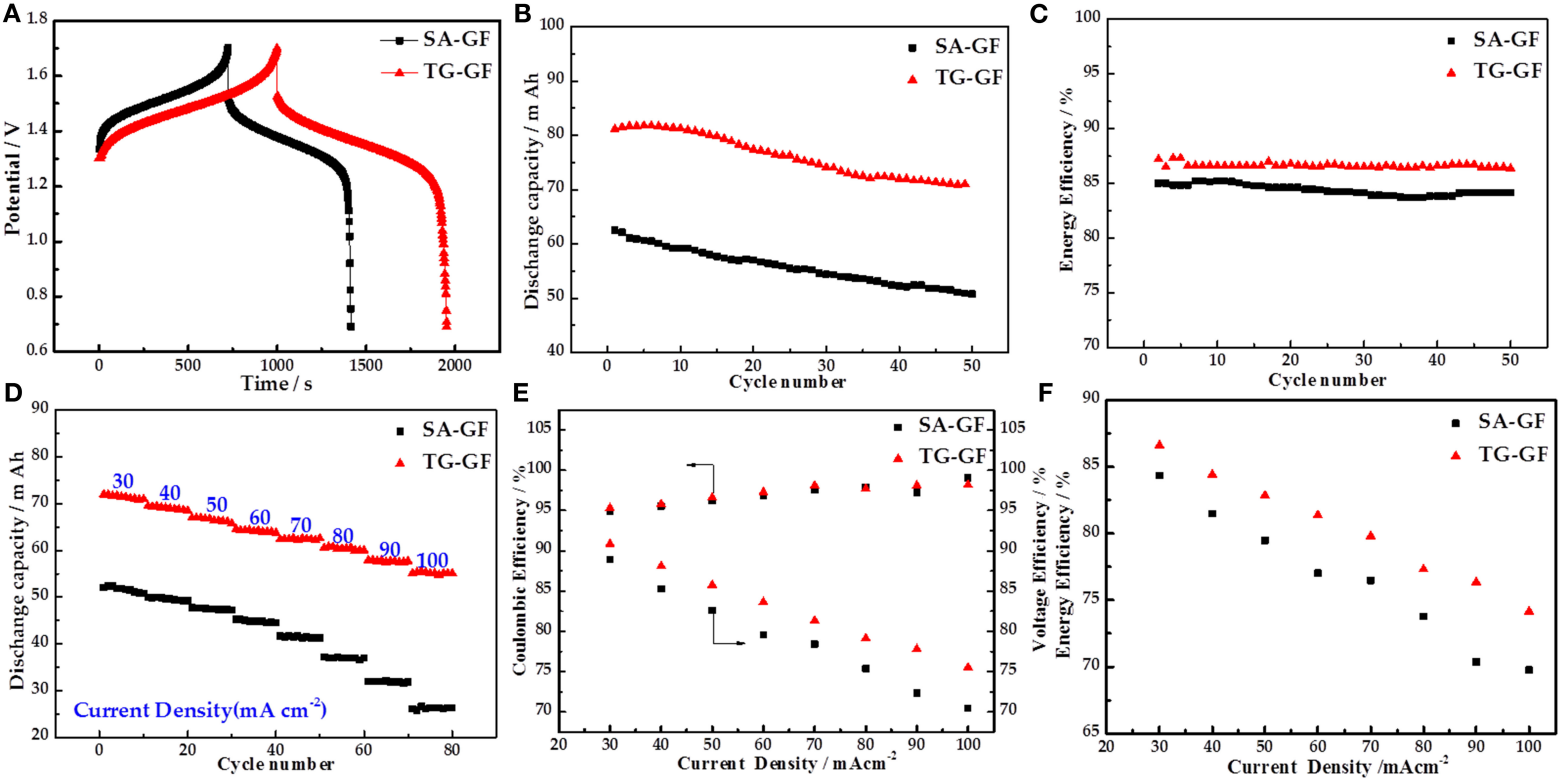
Electrochemical performances of vanadium redox flow battery (VRFB) cells with sulfuric acid-treated graphite felt (SA-GF) and thiourea-grafted graphite felt (TG-GF): **(A)** charge–discharge curves, **(B)** discharge capacity, and **(C)** energy efficiency (EE) of VRFB at the current density of 30 mA cm^−2^; **(D)** discharge capacity, **(E)** coulombic efficiency (CE), voltage efficiency (VE), and **(F)** EE of VRFB at the different current densities.

Figure 3A shows the charge–discharge curves of the cells at 30 mA cm^−2^. Compared with SA-GF-based cell, TG-GF-based cell delivers longer charge–discharge time, lower charge voltage, and higher discharge voltage, which leads to the improvement of the DC and EE. Figure 3B presents the DC dependence on cycle number at 30 mA cm^−2^. TG-GF-based cell shows higher DC than that of SA-GF-based cell. For example, in the first cycle, DC of TG-GF-based cell is 81.2 mA h, 18.7 mA h larger than that of SA-GF-based cell. Meanwhile, the 87.4% DC retention and 87.3% average EE for TG-GF-based cell are 6.0 and 2.5% larger than those of SA-GF-based cell, respectively (Figure 3C).

Figure 3D presents the DC of both cells at different current densities. The DCs of TG-GF-based cell are improved significantly at different current densities. For example, DC of TG-GF-based cell is 55.6 mA h at 100 mA cm^−2^, which is much larger than that of SA-GF-based cell (22.6 mA h). Figures 3E,F show the coulombic efficiency (CE), voltage efficiency (VE), and EE of the cells at different current densities. The CE values for two cells are almost the same, while the VE and EE of TG-GF-based cell are much higher than those of SA-GF-based cell at all current densities, especially at high current density. For example, the VE and EE of TG-GF-based cell are 75.5 and 74.1% at 100 mA cm^−2^, which are 4.9 and 4.4% higher than those of SA-GF-based cell, respectively.

## Conclusions

In order to improve the performance of the electrode, thiourea was grafted onto the surface of SA-GF by thiol-carboxylic acid esterification. Both electrochemical activity and reversibility of the modified electrode toward VO^2+^/${\text{VO}}_{2}^{+}$ redox reaction are improved. Compared with SA-GF-based cell, the cell using TG-GF electrode displays higher DC and VE due to a lower charge transfer resistance, particularly at a high current density.

## Data Availability Statement

The original contributions presented in the study are included in the article/supplementary material, further inquiries can be directed to the corresponding author/s.

## Author Contributions

SW is mainly responsible for experimental operations and drafting paper. XL is mainly responsible for the collecting and processing experimental data. ZG is mainly responsible for collecting information and drafting paper. LW is mainly responsible for designing the experiment and the paper guidance. LD is mainly responsible for reviewing the final manuscript for publication. ZH is mainly responsible for making important modifications to the manuscript.

## Conflict of Interest

The authors declare that the research was conducted in the absence of any commercial or financial relationships that could be construed as a potential conflict of interest.
